# Social Participation and Depressive Symptoms Among Older Adults

**DOI:** 10.1001/jamanetworkopen.2025.30523

**Published:** 2025-09-08

**Authors:** Yuta Takemura, Kosuke Inoue, Koryu Sato, Maho Haseda, Koichiro Shiba, Naoki Kondo

**Affiliations:** 1 Tokyo Bay Urayasu Ichikawa Medical Center, Chiba, Japan; 2 Department of Social Epidemiology, Graduate School of Medicine and School of Public Health, Kyoto University, Kyoto, Japan; 3 Hakubi Center for Advanced Research, Kyoto University, Kyoto, Japan; 4 Faculty of Policy Management, Keio University, Kanagawa, Japan; 5 Department of Epidemiology, Boston University School of Public Health, Boston, Massachusetts

## Abstract

**Importance:**

Previous studies have suggested that social participation helps prevent depression among older adults. However, evidence is lacking about whether the preventive benefits vary among individuals and who would benefit most.

**Objective:**

To examine the sociodemographic, behavioral, and health-related heterogeneity in the association between social participation and depressive symptoms among older adults and to identify the individual characteristics among older adults expected to benefit the most from social participation.

**Design, Setting, and Participants:**

This cohort study included participants aged 65 years or older in the 2016, 2019, and 2022 waves of the Japan Gerontological Evaluation Study (JAGES), a nationwide survey examining social determinants of health and social environment in Japan. All participants were functionally and cognitively independent. Analyses were performed from February 2024 to February 2025.

**Exposures:**

Social participation recorded in the 2019 JAGES. Participation included involvement in hobby groups, sports groups or clubs, volunteer clubs, senior citizen clubs, and neighborhood communities more often than once a month.

**Main Outcomes and Measures:**

The main outcome was depressive symptoms defined as a score of 5 or higher on the Geriatric Depression Scale (GDS; score range: 0-15, with higher scores indicating more severe depressive symptoms) in the 2022 JAGES. After 1:1 propensity score matching on the status of social participation in the 2019 JAGES to adjust for characteristics in the 2016 JAGES, a machine learning–based causal forest algorithm was used to assess the heterogeneity in the association between social participation and depressive symptoms. Conditional average treatment effect (CATE) was estimated for each individual, and the median of the CATE distribution was used as a threshold to categorize participants by their characteristics into the high-benefit group or low-benefit group.

**Results:**

Among the 11 146 propensity score–matched older individuals (mean [SD] age, 72.40 (5.15) years; 5762 men [51.7%]; 15.7% developed depressive symptoms), social participation was associated with reduction in the risk of depressive symptoms (2.2 [95% CI, 0.9-3.5] percentage points). The causal forest model showed heterogeneity across individual characteristics. Compared with the low-benefit group, the high-benefit group were more likely to be older and have lower socioeconomic status (lower educational level and household income). The reduction in risk of depressive symptoms associated with social participation was observed in the high-benefit group (4.1 [95% CI, 1.3-6.9] percentage points) but not in the low-benefit group (0.4 [95% CI, −2.0 to 2.8] percentage points).

**Conclusions and Relevance:**

In this cohort study, the association between social participation and depressive symptoms varied across individual characteristics. Promoting social participation, particularly for older adults with lower socioeconomic status, would play a role in not only improved mental health but also decreased mental health disparities during the global population aging.

## Key Points

**Question:**

Is there heterogeneity in the association between social participation and depressive symptoms by sociodemographic, behavioral, and health-related characteristics among older adults?

**Findings:**

In this cohort study including 11 146 participants in nationwide surveys of older adults in Japan, an association between social participation and depressive symptoms was found. This association was heterogeneous, with social participation associated with a reduced risk of depressive symptoms among older adults and those with lower socioeconomic status.

**Meaning:**

Promoting social participation may be beneficial not only for improving mental health but also for reducing mental health disparities, particularly for older adults with lower socioeconomic status.

## Teaser

This cohort study evaluates the association of involvement in social groups and activities, as well as socioeconomic factors, with depressive symptoms among adults aged 65 years or older.

## Introduction

Depression—a common mental illness linked to suicide, physical illnesses, and economic burden—substantially affects societies worldwide. According to the World Health Organization, approximately 3.8% of the global population experiences depression, including 5.7% of adults older than 60 years, with over 700 000 people dying by suicide annually.^1^ Depression is associated with an increased risk of endocrine, musculoskeletal, and cardiovascular diseases, leading to long-term adverse health outcomes (other than psychiatric disorders) such as premature death.^2,3^ Beyond its health burden, depression imposes substantial economic costs. A Markov model study projected that the undiscounted 10-year cumulative total of all-cause medical costs for individuals with depression from 2023 to 2032 was projected to reach US $309 million by 2032, of which psychiatric care was expected to account for $58 million, representing 19% of the total cost. In addition, the cumulative number of deaths attributable to depression by 2032 was estimated to be 899, suggesting a high fatality rate of 9.8% associated with depression.^4^ Moreover, a pooled cohort analysis of 24 countries showed that low socioeconomic status (eg, lower educational level, lower family income, and not working) was associated with an increased risk of depression (hazard ratio, 1.34).^5^ Given the adverse health and economic impacts of depression and socioeconomic inequality in this mental health disorder, building effective management and prevention of depression is urgently needed.

Social participation is key to preventing depression.^6^ Ample evidence has shown that participating in the activities of formal and informal societal groups was associated with reduced risk of depressive symptoms among older adults.^7,8,9^ Because the occurrence of depression involves multifaceted mechanisms, the potential benefits of social participation for mental health likely vary by individual factors, including gender, age, socioeconomic status, and health conditions. Identifying individual differences and understanding the characteristics of those who may experience greater preventive benefits are crucial for building a mentally resilient society. Despite growing interest, it remains unclear who derives the most mental health benefit from social participation. Conventional subgroup analyses are typically limited to a few covariates when setting an arbitrary threshold for continuous variables (eg, subgroup analysis by age [>75 vs ≤75 years] or gender [men vs women]). In contrast, machine learning methods can simultaneously account for a broader range of continuous and categorical variables (eg, age, gender, and income), which enable more refined assessments of heterogeneity by capturing a complex, high-dimensional, and nonlinear interplay across individual-level characteristics.^10,11,12^

To address this knowledge gap, we aimed to examine the sociodemographic, behavioral, and health-related heterogeneity in the association between social participation and depressive symptoms among older adults, and to identify the individual characteristics of older adults expected to benefit the most from social participation. We used a machine learning–based causal forest algorithm with data obtained from the Japan Gerontological Evaluation Study (JAGES) to inform precision public health strategies for mental resilience during global population aging.

## Method

### Design and Participants

We used longitudinal cohort data from the 2016, 2019, and 2022 waves of JAGES, which is a nationwide gerontological survey examining social determinants of health and social environment in Japan. The Ethical Committee at the National Centre for Geriatrics and Gerontology, Chiba University, and Kyoto University approved the JAGES study protocol. Participants were informed that their involvement was voluntary and that the return of questionnaires indicated giving their consent. We followed the Strengthening the Reporting of Observational Studies in Epidemiology (STROBE) reporting guideline.

The 2016 JAGES, conducted between October 2016 and January 2017, enrolled 279 661 older adults aged 65 years or older. Participants were selected from the municipal long-term care insurance registries in 39 municipalities across 18 prefectures, with some municipalities conducting a census of all eligible individuals. The sample included individuals followed up from the 2013 JAGES as well as those newly selected through random sampling from municipalities that were not included in the 2013 survey. In the random sampling process, self-administered questionnaires were mailed to all eligible participants, and internet-based questionnaires were sent to approximately 11% of the total target population. The JAGES dataset included older adults who were independent in activities of daily living; therefore, participants who had been certified as requiring long-term care during the study period were excluded (ie, all participants were functionally and cognitively independent). Response rates were 70.0% in 2016, 68.7% in 2019, and 66.2% in 2022. The 2016 JAGES was used as the prebaseline survey, while the 2019 JAGES served as the baseline survey.

### Exposure

The exposure variable in this study was social participation, as recorded in the 2019 JAGES. Social participation was categorized into 5 distinct groups: hobby groups, sports groups or clubs, volunteer clubs, senior citizen clubs, and neighborhood communities.^8,13^ Senior citizen clubs provide older adults with opportunities to engage in diverse activities, including sports, hobbies, cultural pursuits, and the arts. Neighborhood communities are resident-led organizations focused on local governance and fostering shared community interests.

JAGES participants were asked, “How often do you participate in the following groups or clubs?” and selected 1 of the following responses: almost every day, two to three times a week, once or twice a month, a few times a year, or never. Based on these responses, we created a binary composite social participation variable, where respondents who participated in any groups or clubs more often than once a month were assigned a score of 1, while those who never or a few times a year participated in groups or clubs were assigned a score of 0, following a previous study.^8^

### Outcome

The Japanese short version of the Geriatric Depression Scale (GDS), a widely used tool for assessing depressive symptoms in older adults, was administered to JAGES participants. The GDS consists of 15 binary questions, each requiring a yes or no response, with total scores ranging from 0 to 15 (eTable 1 in Supplement 1).^14^ Higher scores indicate more severe depressive symptoms.^15^ In this study, the main outcome was depressive symptoms, defined as a GDS score of 5 or higher, in the 2022 JAGES. This outcome was based on a prior validation study that reported a sensitivity of 92% and a specificity of 81% compared with the gold standard clinical diagnosis using the Structured Clinical Interview for *Diagnostic and Statistical Manual of Mental Disorders* (*Third Edition Revised*).^16^ Individuals with a GDS score of 5 or higher were coded as 0, and those with a GDS score lower than 5 were coded as 1. Details in prebaseline covariates in the 2016 JAGES are described in the eMethods in Supplement 1.

### Propensity Score Matching

We applied 1:1 propensity score matching (PSM) without replacement to match participants based on their social participation in the 2019 JAGES, adjusting for potential confounders (ie, all prebaseline covariates, including social participation in the 2016 JAGES). Propensity scores for social participation were calculated using a logistic regression model. An absolute standardized mean difference (SMD) of less than 0.1 indicated a successful balance between matched groups.^17^ To reduce bias from missing data, missing values were imputed using chained random forests under the assumption of data missing at random,^18^ prior to conducting PSM.

### Statistical Analysis

First, using the grf package in R (R Project for Statistical Computing), we applied a machine learning causal forest algorithm to the randomly selected half of matched sample (training data) to develop a model estimating the reduction in depressive symptoms with social participation at the individual level.^19^ In the causal forest model, an ensemble of 2000 trees was constructed using all covariates included in PSM. Model calibration was assessed by fitting the best linear prediction and estimating the subgroup-specific average treatment effect (ATE) according to the quintiles of the conditional average treatment effect (CATE) estimated from the causal forest model. The subgroup-specific ATEs were estimated using augmented inverse probability weighting regression adjusted for all prebaseline covariates, including social participation in 2016. The CATE can be interpreted as the estimated change in the risk of depressive symptoms associated with social participation that is conditional on individual characteristics; that is, the positive CATE estimates would indicate decreased risk of depressive symptoms associated with social participation. The variable importance was estimated based on a simple weighted sum of the number of split times of each variable in the causal forest model. Further details on the causal forest algorithm are available elsewhere.^19,20^

Next, we divided the test data (remaining half of the propensity score–matched sample) into 2 subgroups using the threshold of the median of the CATE distribution (high-benefit group [CATE above the median] vs low-benefit group [CATE at or below the median]). We compared the distribution of the baseline characteristics between the high-benefit group and the low-benefit group. We also compared the characteristics of participants across the quintiles of the estimated CATE from the causal forest model.

Furthermore, we investigated the association between social participation and depressive symptoms in the high-benefit group and the low-benefit group using augmented inverse probability weighting estimator. The 95% CIs of each estimate and their difference were calculated using nonparametric bootstrap resampling (1000 iterations) with the percentile method.

We conducted several additional analyses. First, because 45.1% of older adults in the 2016 and 2019 JAGES did not participate in the 2022 JAGES, we applied inverse probability of censoring weights for those lost to follow-up in our analysis. Second, we conducted the complete case analysis instead of imputing missing values. Third, we reanalyzed the data using 2 alternative cutoffs to define social participation: at least once per year and at least once per week.

All analyses were performed from February 2024 to February 2025. A 95% CI that did not cross 0 was considered statistically significant. We used R, version 4.4.0 (R Project for Statistical Computing).

## Results

### Characteristics of Study Participants

**Figure 1.**
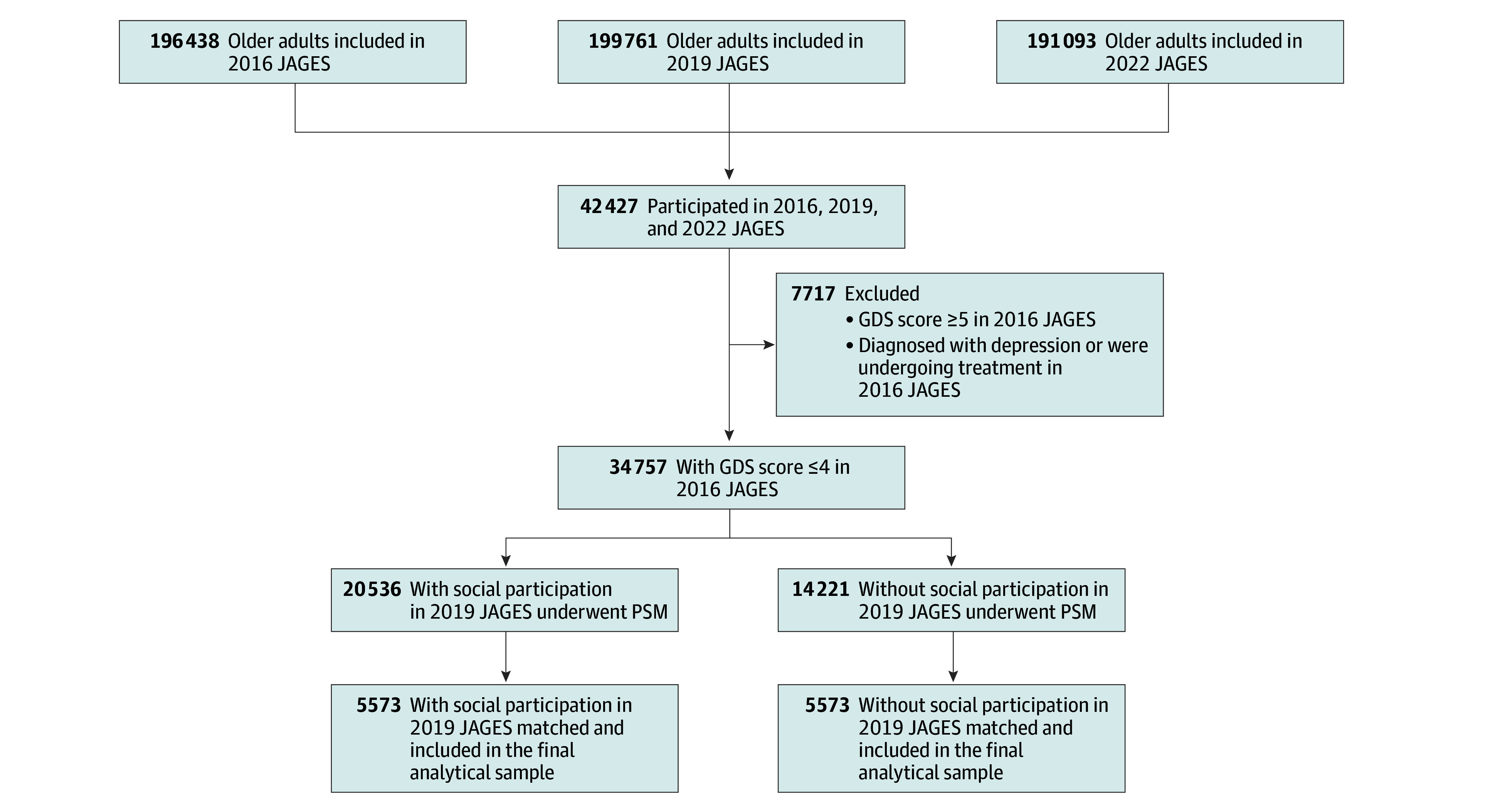
Flow Diagram of Study Sample Individuals with certification for long-term care were not included in the sample because the Japan Gerontological Evaluation Study (JAGES) dataset targets community-dwelling older adults with independent activities of daily living. GDS indicates Geriatric Depression Scale; PSM, propensity score matching.

Among 77 283 older adults who participated in both 2016 and 2019 JAGES, 54.9% (n = 42 427) continued to participate in the 2022 survey (eTable 2 in Supplement 1). Among the 34 757 participants without depressive symptoms (GDS score ≤4 and no depression history) in the 2016 JAGES before PSM (Figure 1), there were 18 074 women (52.0%) and 16 683 men (48.0%) with a mean (SD) age of 72.55 (5.09) years. At least 1 prebaseline variable was missing in 61.2% (n = 25 975) of participants. Compared with individuals without social participation (14 221 [40.9%]), those with social participation (20 536 [59.1%]) were more likely to be women, retired, with a high educational level, a nonsmoker, and have frequent interaction with friends (Table 1). After PSM, the 2 groups (with vs without social participation) were well balanced across all baseline covariates, with SMD less than 0.1 for all covariates.^21^

**Table 1.** Baseline Characteristics of Participants According to Social Participation in 2019, Before and After Propensity Score Matching

Variable	Participants, No. (%)					
	Before PSM			After PSM		
	Without social participation in 2019 (n = 14 221)	With social participation in 2019 (n = 20 536)	SMD^a^	Without social participation in 2019 (n = 5573)	With social participation in 2019 (n = 5573)	SMD^a^
Age, mean (SD), y	72.33 (5.28)	72.69 (4.95)	0.071	72.41 (5.23)	72.40 (5.06)	0.002
Gender						
Men	7732 (54.4)	8972 (43.7)	0.215	2907 (52.2)	2858 (51.3)	0.018
Women	6489 (45.6)	11 564 (56.3)		2666 (47.8)	2715 (48.7)	
BMI, mean (SD)	23.11 (3.12)	22.85 (2.90)	0.088	23.13 (3.06)	23.06 (2.97)	0.025
Work status						
Working	5422 (38.1)	5627 (27.4)	0.230	1994 (35.8)	2010 (36.1)	0.019
Retired	7969 (56.0)	13 519 (65.8)		3249 (58.3)	3211 (57.6)	
Nonworking	830 (5.8)	1390 (6.8)		330 (5.9)	352 (6.3)	
Marital status						
Married	11 424 (80.3)	16 218 (79.0)	0.116	4476 (80.3)	4471 (80.2)	0.024
Widowed	1873 (13.2)	3358 (16.4)		786 (14.1)	797 (14.3)	
Divorced	516 (3.6)	563 (2.7)		168 (3.0)	179 (3.2)	
Unmarried	336 (2.4)	343 (1.7)		119 (2.1)	106 (1.9)	
Other^b^	72 (0.5)	54 (0.3)		24 (0.4)	20 (0.4)	
Educational level, y						
<6	52 (0.4)	43 (0.2)	0.227	22 (0.4)	17 (0.3)	0.025
6-9	4316 (30.3)	4383 (21.3)		1528 (27.4)	1497 (26.9)	
10-12	6161 (43.3)	9297 (45.3)		2432 (43.6)	2456 (44.1)	
≥13	3641 (25.6)	6697 (32.6)		1566 (28.1)	1572 (28.2)	
Other^b^	51 (0.4)	116 (0.6)		25 (0.4)	31 (0.6)	
Annual household income, US $						
<18 386	2959 (20.8)	3346 (16.3)	0.121	1061 (19.0)	1093 (19.6)	0.030
18 386-36 771	6126 (43.1)	9020 (43.9)		2435 (43.7)	2353 (42.2)	
>36 771	5136 (36.1)	8170 (39.8)		2077 (37.3)	2127 (38.2)	
Medical history						
Hypertension	6441 (45.3)	8347 (40.6)	0.129	2451 (44.0)	2430 (43.6)	0.028
Stroke	133 (0.9)	146 (0.7)		45 (0.8)	34 (0.6)	
Heart diseases	580 (4.1)	771 (3.8)		223 (4.0)	220 (3.9)	
Diabetes	786 (5.5)	1006 (4.9)		296 (5.3)	288 (5.2)	
Hyperlipidemia	742 (5.2)	1483 (7.2)		322 (5.8)	324 (5.8)	
Other^c^	5539 (38.9)	8783 (42.8)		2236 (40.1)	2277 (40.9)	
Smoking status						
Nonsmoker	7810 (54.9)	13 342 (65.0)	0.219	3210 (57.6)	3246 (58.2)	0.019
Past smoker	4771 (33.5)	5717 (27.8)		1809 (32.5)	1760 (31.6)	
Current smoker	1640 (11.5)	1477 (7.2)		554 (9.9)	567 (10.2)	
Alcohol consumption						
Nondrinker	6624 (46.6)	9964 (48.5)	0.101	2604 (46.7)	2624 (47.1)	0.011
Past drinker	1446 (10.2)	1503 (7.3)		499 (9.0)	509 (9.1)	
Current drinker	6151 (43.3)	9069 (44.2)		2470 (44.3)	2440 (43.8)	
IADL						
Independence	13 144 (92.4)	19 653 (95.7)	0.139	5215 (93.6)	5220 (93.7)	0.004
Dependence	1077 (7.6)	883 (4.3)		358 (6.4)	353 (6.3)	
Unmarried or living alone						
No	11 020 (77.5)	15 735 (76.6)	0.021	4337 (77.8)	4326 (77.6)	0.005
Yes	3201 (22.5)	4801 (23.4)		1236 (22.2)	1247 (22.4)	
Poor interaction with children						
No	10 306 (72.5)	15 272 (74.4)	0.043	4079 (73.2)	4106 (73.7)	0.011
Yes	3915 (27.5)	5264 (25.6)		1494 (26.8)	1467 (26.3)	
Poor interaction with relatives						
No	7481 (52.6)	10 728 (52.2)	0.007	2924 (52.5)	2982 (53.5)	0.021
Yes	6740 (47.4)	9808 (47.8)		2649 (47.5)	2591 (46.5)	
Poor interaction with friends						
No	8334 (58.6)	16 941 (82.5)	0.543	3857 (69.2)	3866 (69.4)	0.004
Yes	5887 (41.4)	3595 (17.5)		1716 (30.8)	1707 (30.6)	
Social participation in 2016						
No	11 048 (77.7)	2421 (11.8)	1.770	2417 (43.4)	2417 (43.4)	<0.001
Yes	3173 (22.3)	18 115 (88.2)		3156 (56.6)	3156 (56.6)	

Abbreviations: BMI, body mass index (calculated as weight in kilograms divided by height in meters squared); IADL, instrumental activities of daily living; PSM, propensity score matching; SMD, standardized mean difference.

^a^ One-to-one pair matching was performed using nearest-neighbor matching without replacement, with a caliper equal to 0.1 of the SD of the logit of the propensity score. SMD less than 0.1 was considered to be a successful balance between the 2 groups.

^b^ No additional information provided.

^c^ Other medical history includes pulmonary diseases, gastrointestinal diseases, kidney diseases, musculoskeletal disorders, trauma (eg, falls and fractures), cancer, hematologic and immunologic disorders, ophthalmologic conditions, and otologic conditions.

### Heterogeneity in the Association Between Social Participation and Depressive Symptoms

A total of 11 146 participants (mean [SD] age, 72.40 [5.15] years; 5384 women [48.3%] and 5762 men [51.7%]) were propensity score–matched. Over the 3-year follow-up of these propensity score–matched participants, 1751 (15.7%) developed depressive symptoms (16.8% [938 of 5573] without social participation vs 14.6% [813 of 5573] with social participation). Social participation was associated with the reduction in the risk of depressive symptoms among the entire sample of propensity score-matched participants (2.2 [95% CI, 0.9-3.5] percentage points).

**Figure 2.**
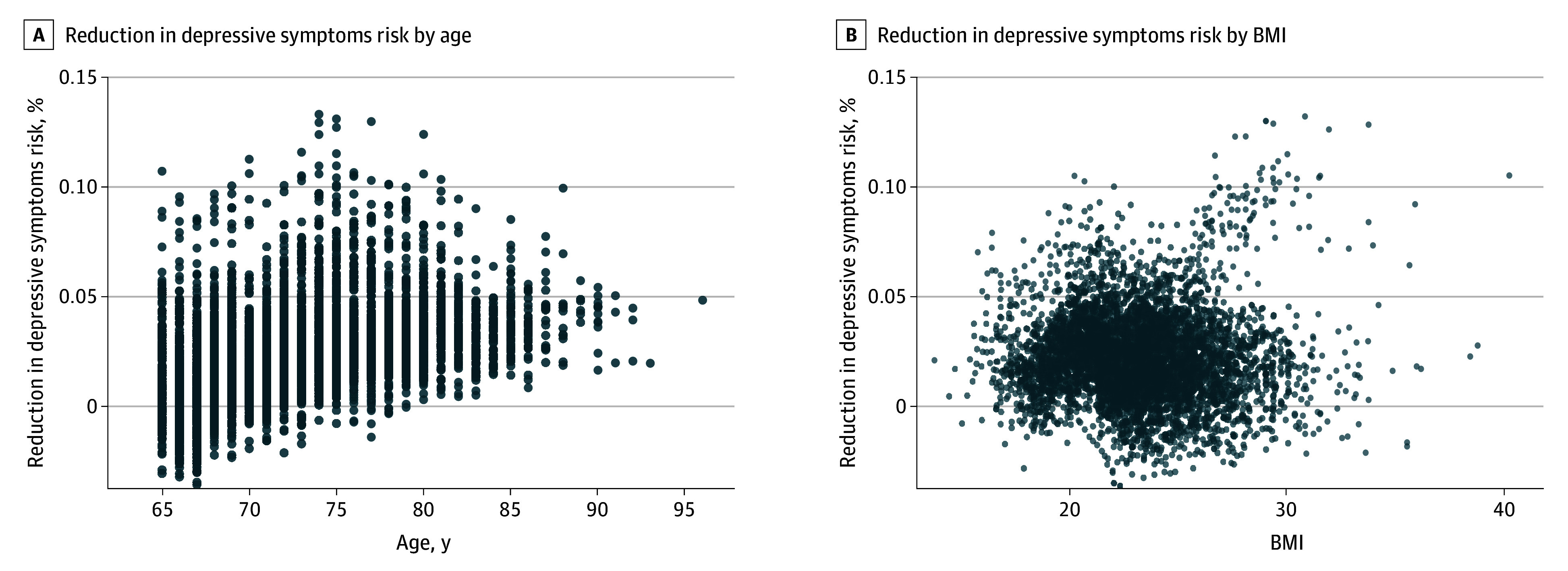
Estimated Reduction in the Risk of Depressive Symptoms in 2022 by Social Participation in 2019 by Age and Body Mass Index (BMI) BMI was calculated as weight in kilograms divided by height in meters squared.

Our causal forest model revealed sociodemographic, behavioral, and health-related heterogeneity in the association between social participation and depressive symptoms, indicating that this association varied across individuals. The median (IQR) of the estimated CATE was 2.1 (1.0-3.3) percentage points. We found the monotonic increase in group-specific ATEs by quintiles of the CATE, supporting the good calibration of the model (eFigure 1 in Supplement 1). Body mass index (BMI; calculated as weight in kilograms divided by height in meters squared) and age showed high variable importance of the causal forest model, followed by responses to some GDS questions in the 2016 JAGES (eFigure 2 in Supplement 1). When we plotted the estimated CATE by BMI and age, we observed a dose-response association between older age and higher CATE, while the patterns for BMI were not clear (Figure 2).

### Characteristics of Participants in the High-Benefit Group

Participants in the high-benefit group were primarily characterized by the following prebaseline covariates (SMD >0.2): older age, nonworking status, being widowed or unmarried or living alone, educational level of less than 9 years, annual household income less than US $18 386, and past smoker status (Table 2). In particular, the high-benefit group showed a higher mean (SD) age than the low-benefit group (74.70 [5.24] vs 70.20 [4.09] years; SMD, 0.957). In terms of socioeconomic status (SES), a greater proportion of individuals in the high-benefit group were retired (62.2% [1733] vs 54.4% [1516]; SMD, 0.232) and fewer were married (72.0% [2007] vs 89.5% [2494]; SMD, 0.514) compared with the low-benefit group. Additionally, 870 participants (31.2%) in the high-benefit group were either unmarried or living alone compared with 345 participants (12.4%) in the low-benefit group. The high-benefit group comprised 919 individuals (33.0%) with less than 9 years of education compared with 642 individuals (23.1%) in the low-benefit group. Furthermore, a higher proportion of participants in the high-benefit group had an annual household income less than $18 386 compared with the low-benefit group (24.2% [673] vs 14.2% [395]; 1156 participants (41.5%) in the low-benefit group earned more than $36 771 per year. The prevalence of past smoking was also higher in the high-benefit group than in the low-benefit group (38.2% [1065] vs 27.4% [764]). Similar patterns were observed when we compared the characteristics across the quintiles of the estimated CATE from the causal forest model (eTable 3 in Supplement 1).

**Table 2.** Comparison of Characteristics in the High-Benefit vs Low-Benefit Groups

Variable	Participants, No. (%)		SMD
	Low-benefit group (n = 2787)^a^	High-benefit group (n = 2786)^a^	
Age, mean (SD), y	70.20 (4.09)	74.70 (5.24)	0.957
Gender			
Men	1400 (50.2)	1514 (54.3)	0.082
Women	1387 (49.8)	1272 (45.7)	
BMI, mean (SD)	23.30 (3.08)	22.98 (2.96)	0.105
Work status			
Working	1137 (40.8)	844 (30.3)	0.232
Retired	1516 (54.4)	1733 (62.2)	
Nonworking	134 (4.8)	209 (7.5)	
Marital status			
Married	2494 (89.5)	2007 (72.0)	0.514
Widowed	148 (5.3)	619 (22.2)	
Divorced	80 (2.9)	87 (3.1)	
Unmarried	60 (2.2)	61 (2.2)	
Other^b^	5 (0.2)	12 (0.4)	
Educational level, y			
<6	2 (0.1)	14 (0.5)	0.233
6-9	640 (23.0)	905 (32.5)	
10-12	1327 (47.6)	1126 (40.4)	
≥13	801 (28.7)	726 (26.1)	
Other^b^	17 (0.6)	15 (0.5)	
Annual household income, US $			
<18 386	395 (14.2)	673 (24.2)	0.268
18 386-36 771	1236 (44.3)	1183 (42.5)	
>36 771	1156 (41.5)	930 (33.4)	
Medical history			
Hypertension	1123 (40.3)	1352 (48.5)	0.168
Stroke	21 (0.8)	18 (0.6)	
Heart diseases	127 (4.6)	100 (3.6)	
Diabetes	152 (5.5)	140 (5.0)	
Hyperlipidemia	159 (5.7)	144 (5.2)	
Other^c^	1205 (43.2)	1032 (37.0)	
Smoking status			
Nonsmoker	1626 (58.3)	1539 (55.2)	0.311
Past smoker	764 (27.4)	1065 (38.2)	
Current smoker	397 (14.2)	182 (6.5)	
Alcohol consumption			
Nondrinker	1332 (47.8)	1250 (44.9)	0.103
Past drinker	220 (7.9)	299 (10.7)	
Current drinker	1235 (44.3)	1237 (44.4)	
IADL			
Independence	2653 (95.2)	2550 (91.5)	0.148
Dependence	134 (4.8)	236 (8.5)	
Unmarried or living alone			
No	2442 (87.6)	1916 (68.8)	0.469
Yes	345 (12.4)	870 (31.2)	
Poor interaction with children			
No	2068 (74.2)	2014 (72.3)	0.043
Yes	719 (25.8)	772 (27.7)	
Poor interaction with relatives			
No	1405 (50.4)	1550 (55.6)	0.105
Yes	1382 (49.6)	1236 (44.4)	
Poor interaction with friends			
No	2003 (71.9)	1815 (65.1)	0.145
Yes	784 (28.1)	971 (34.9)	
Social participation in 2016			
No	1276 (45.8)	1144 (41.1)	0.095
Yes	1511 (54.2)	1642 (58.9)	

Abbreviations: BMI, body mass index (calculated as weight in kilograms divided by height in meters squared); IADL, instrumental activities of daily living; SMD, standardized mean difference.

^a^ The high-benefit group includes individuals with an estimated conditional average treatment effect (CATE) above the median, while the low-benefit group includes those with a CATE at or below the median (IQR) of 2.1 (1.0-3.3) percentage points.

^b^ No additional information provided.

^c^ Other medical history includes pulmonary diseases, gastrointestinal diseases, kidney diseases, musculoskeletal disorders, trauma (eg, falls and fractures), cancer, hematologic and immunologic disorders, ophthalmologic conditions, and otologic conditions.

### Association Between Social Participation and Depressive Symptoms in the High-Benefit and Low-Benefit Groups

Social participation was associated with decreased risk of depressive symptoms in the high-benefit group (4.1 [95% CI, 1.3-6.9] percentage points) but not the low-benefit group (0.4 [95% CI, −2.0 to 2.8] percentage points) (Table 3). The absolute difference in risk reduction in depressive symptoms between the high-benefit and low-benefit groups was 3.7 (95% CI, −0.2 to 7.2) percentage points.

**Table 3.** Association Between Social Participation in 2019 and Depressive Symptoms in the High-Benefit vs Low-Benefit Groups

	Risk reduction of depressive symptoms, percentage points (95% CI)^a^	
	High-benefit group	Low-benefit group
Main analysis		
Without social participation in 2019	1 [Reference]	1 [Reference]
With social participation in 2019	4.1 (1.3 to 6.9)	0.4 (−2.0 to 2.8)
Additional analyses		
IPCW for loss-follow-up or death		
Without social participation in 2019	1 [Reference]	1 [Reference]
With social participation in 2019	4.0 (1.2 to 6.8)	0.4 (−2.0 to 2.7)
Complete case analysis		
Without social participation in 2019	1 [Reference]	1 [Reference]
With social participation in 2019	2.6 (−1.9 to 7.2)	0.6 (−2.7 to 3.9)
Different threshold to define social participation		
At least once per y in 2019		
Without social participation in 2019	1 [Reference]	1 [Reference]
With social participation in 2019	7.1 (3.8 to 10.3)	−0.1 (−2.8 to 2.6)
At least once per wk in 2019		
Without social participation in 2019	1 [Reference]	1 [Reference]
With social participation in 2019	3.5 (0.4 to 6.6)	0.4 (−2.2 to 3.0)

Abbreviation: IPCW, inverse probability of censoring weights.

^a^ The augmented inverse probability weight estimator was applied to estimate risk reduction in depressive symptoms due to social participation. The 95% CIs were constructed using nonparametric bootstrap resampling (1000 iterations) with the percentile method.

### Additional Analyses

We found consistent results when we applied inverse probability of censoring weights, when we conducted a complete case analysis, and when we redefined social participation under the annual threshold and the weekly threshold (Table 3). For example, social participation was associated with a decreased risk of depressive symptoms in the high-benefit group (7.1 [95% CI, 3.8-10.3] percentage points for participation at least once per year; 3.5 [95% CI, 0.4-6.6] percentage points for participation at least once per week), whereas no such association was observed in the low-benefit group under either definition.

## Discussion

In this longitudinal cohort study of older adults in Japan, we found heterogeneity in the association between social participation and depressive symptoms. In particular, older age, nonworking status, being unmarried or living alone, educational level of less than 9 years, annual household income less than $18 386, and past smoker status were associated with mental health benefits from social participation. The high-benefit group showed a higher reduction (4.1 percentage points) in depressive symptoms associated with social participation than the low-benefit group, which is a meaningful reduction from a public health perspective, although the difference was not statistically significant.

Ample evidence has shown that social participation mitigates psychological distress by broadening social networks, enhancing social support from friends, and providing a sense of purpose in life.^22,23,24^ While some studies have suggested the larger mental health benefits of social participation for older vs younger adults,^25^ whether such age-based differences exist even among older adults remains unclear. Our results generate the hypothesis that the oldest adults receive the most pronounced mental health benefits from social participation, which is likely to become increasingly valuable given the aging of the population worldwide.

We observed heterogeneity in the association between social participation and a reduced risk of depressive symptoms. Although the differences across subgroups included the null in their 95% CIs, the intervals were asymmetrically skewed toward the positive direction, and the point estimates were also skewed toward the positive direction. These findings support the potential utility of a high-benefit approach in promoting social participation. Individuals in the high-benefit group were likely to have lower SES, such as lower educational level, lower household income, and being unmarried or living alone. They also showed a higher prevalence of past smoking status, which is associated with lower SES.^26^ Such individuals with lower SES are likely to face difficulties in engaging in social participation due to limited transportation options, financial constraints, and restricted access to information.^27,28^ Addressing the challenges faced by individuals with lower SES and promoting the creation of environments that facilitate their engagement in social participation may contribute to maximizing the mental health benefits derived from social participation.

Given the high prevalence of depression among people with lower SES (including lower educational level and lower household income^5^) and with social isolation (including living alone^7^), mental health disparities associated with SES represent a substantial issue in the field of social epidemiology. Although social interventions such as social participation are generally implemented using a population-based approach, this approach may not effectively reach individuals with lower SES, potentially exacerbating existing mental health disparities.^29^ In this context, our findings underscore the importance of implementing targeted interventions that promote social participation among older adults with low SES, in addition to conventional population- and community-level health promotion strategies, to reduce mental health disparities associated with sociodemographic factors in later life.

### Limitations

Our study has several limitations. First, while we applied the PSM method to balance the distribution of measured covariates between social participation and depressive symptoms, our results may still be affected by confounding bias due to unmeasured covariates. For example, although individuals certified as requiring long-term care were not included in the JAGES dataset, it is possible that some participants received daily living support from informal caregivers, which may affect the probability of social participation and the risk of depressive symptoms. In addition, PSM was performed on the overall cohort data; thus, the distribution of measured covariates may not be balanced within each subgroup. However, the same covariates used for PSM were included as covariates in the causal forest model. Therefore, in theory, the estimated CATE can be considered to satisfy the assumption of conditional exchangeability. Second, social participation was self-reported and thus might be misclassified.

Third, we defined social participation as a single exposure and did not differentiate its various forms, such as involvement in social clubs, volunteer activities, and sports groups. Future studies are needed to investigate the heterogeneity in the mental health benefits of each type of social participation. Fourth, our results might be biased due to missing values because we applied single imputation via random forest given the computational intensity of multiple imputation. However, we found that the results were qualitatively consistent in the complete case analysis. Therefore, missing data were handled using single imputation by chained random forests to reduce computational demands. Finally, as the JAGES dataset only included older respondents in Japan who remained functionally and cognitively independent across all 3 survey waves, the generalizability and transportability of our results to different populations are limited, particularly given the cultural differences in social participation across countries.

## Conclusions

We found heterogeneity in the association between social participation and decreased risk of depressive symptoms among older adults. In particular, people of older age, with lower SES (eg, lower educational level and lower household income), and who were unmarried or living alone were more likely to receive mental health benefits from social participation. It is imperative to create an environment that increases opportunities for social participation, particularly for these socioeconomically marginalized populations. Social participation would play a role in not only improved population mental health but also mitigate the mental health disparities by SES during the global population aging.

## Notes

Supplement 1. **eMethods.** Pre-Baseline Covariates Ascertainment **eFigure 1.** Quintile Plot of Causal Forest Model **eFigure 2.** Variable Importance of the Causal Forest Model **eTable 1.** Geriatric Depression Scale 15 **eTable 2.** Baseline Characteristics of Participants in Both 2016 and 2019, and Those Followed From 2016, 2019, to 2022 **eTable 3.** Comparison of Characteristics Among Five Subgroups Based on Quintiles of the CATE Distribution
eReferences

Supplement 2.
Data Sharing Statement
