# Supplementary material for: Social Participation and Depressive Symptoms Among Older Adults
Source: JAMA Netw Open. 2025 Sep 8;8(9):e2530523. doi: 10.1001/jamanetworkopen.2025.30523 (PMC12418130; doi:10.1001/jamanetworkopen.2025.30523)
Supplement: Supplement 1. — eMethods. Pre-Baseline Covariates Ascertainment eFigure 1. Quintile Plot of Causal Forest Model eFigure 2. Variable Importance of the Causal Forest Model eTable 1. Geriatric Depression Scale 15 eTable 2. Baseline Characteristics of Participants in Both 2016 and 2019, and Those Followed From 2016, 2019, to 2022 eTable 3. Comparison of Characteristics Among Five Subgroups Based on Quintiles of the CATE Distribution eReferences [file jamanetwopen-e2530523-s001.pdf]

Supplemental Online Content

Takemura Y, Inoue K, Sato K, Haseda M, Shiba K, Kondo N. Social participation and depressive symptoms among older adults. *JAMA Netw Open*. 2025;8(9):e2530523. doi:10.1001/jamanetworkopen.2025.30523

eMethods. Pre-Baseline Covariates Ascertainment

eFigure 1. Quintile Plot of Causal Forest Model

eFigure 2. Variable Importance of the Causal Forest Model

eTable 1. Geriatric Depression Scale 15

eTable 2. Baseline Characteristics of Participants in Both 2016 and 2019, and Those Followed From 2016, 2019, to 2022

eTable 3. Comparison of Characteristics Among Five Subgroups Based on Quintiles of the CATE Distribution

eReferences

This supplemental material has been provided by the authors to give readers additional information about their work.

© 2025 Takemura Y et al. *JAMA Network Open*

eMethods. Pre-baseline Covariates Ascertainment

At the pre-baseline survey of 2016, participants provided sociodemographic data, including age (in years), gender (men, women), body mass index (BMI), educational attainment (≤6 years, 6-9 years, 10-12 years, ≥13 years, others), marital status (married, widowed, divorced, unmarried, and others), annual household income (≤18,386 US dollars, 18,386 – 36,771 US dollars, ≥36,771 US dollars per year), smoking status (current, former, never), drinking status (current, former, never), instrumental activities of daily living (IADL), history of previous illnesses, interaction with others (i.e., children, relatives, and friends), and presence or absence of cohabitant. IADL was assessed through five binary questions regarding participants' ability to: (i) use transportation, (ii) prepare meals, (iii) shop, (iv) pay bills, and (v) manage finances independently.[1] Each activity was assigned 1 point, with a total possible score of 5; a score of less than 5 indicates some level of functional impairment. The history of past diseases, such as hypertension, heart diseases, stroke, diabetes, hyperlipidemia, and others (musculoskeletal disorders, injuries, cancers, hematologic and immune system disorders, ophthalmologic diseases, and otologic diseases), was self-reported by participants. Interaction with others and the presence or absence of a cohabitant were defined based on the following criteria: (i) individuals who did not live with their children or lacked emotional or instrumental social support from them, (ii) those without immediate family members providing emotional or instrumental social support, (iii) individuals who had face-to-face contact with friends less than once a month or lacked friends capable of providing emotional or instrumental social support, and (iv) participants who were not married or lived alone.[2] Information on social participation in 2016 was also collected.

© 2025 Takemura Y et al. *JAMA Network Open*

eFigure 1. Quintile plot of causal forest model.

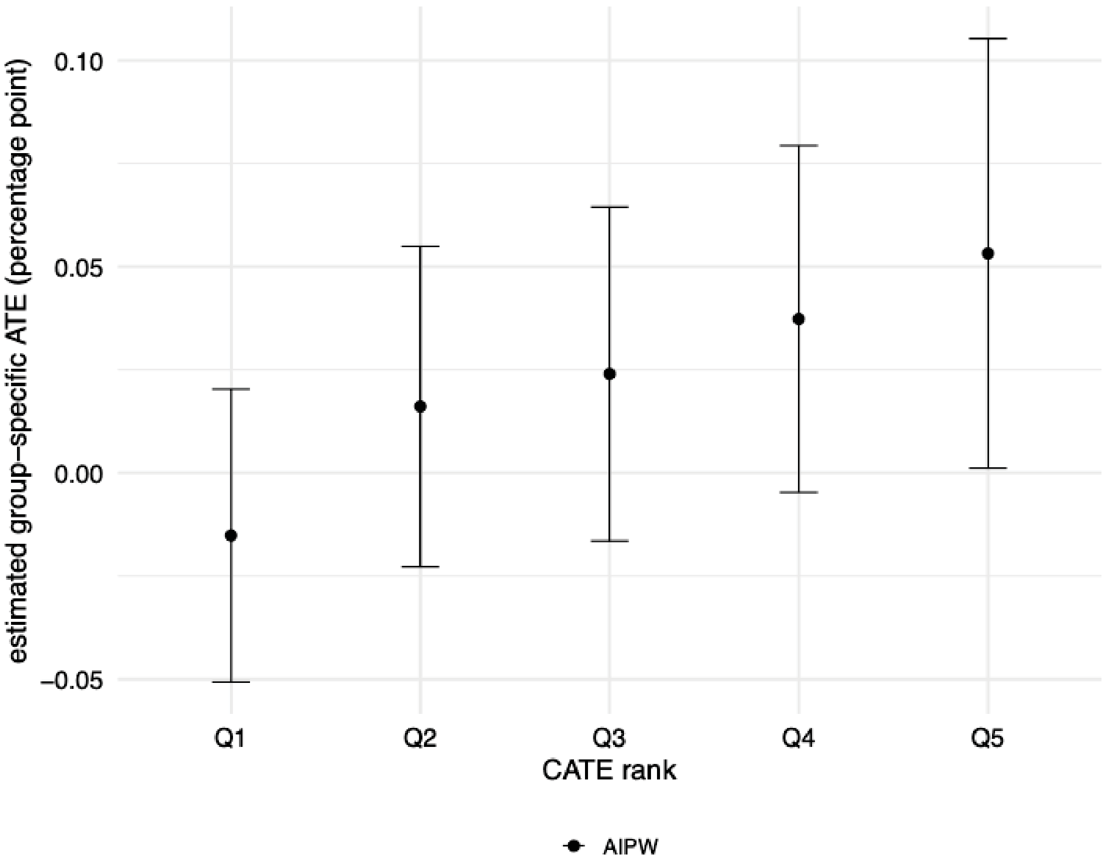

Conditional average treatment effects (CATE) of social participation in 2019 on depressive symptoms in 2022 are ranked by the predicted treatment effects derived from the causal forest model (X-axis). The Y-axis represents the group-specific average treatment effect (ATE).

Group-specific ATEs were estimated using augmented inverse probability weighting (AIPW) regression adjusted for baseline covariates (age, gender, BMI, work status, marital status, educational attainment, household income, medical history, smoking, alcohol consumption, instrumental activities of daily living (IADL), the status of social participation in 2016, unmarried or living alone, and poor interaction with children, relatives, and/or friends).

In the best linear prediction, the coefficient for the mean forest prediction was 0.90 (p-value < 0.01) and the coefficient for the out-of-bag predicted association was 0.64 (p-value = 0.103).

© 2025 Takemura Y et al. *JAMA Network Open*

eFigure 2. Variable importance of the causal forest model.

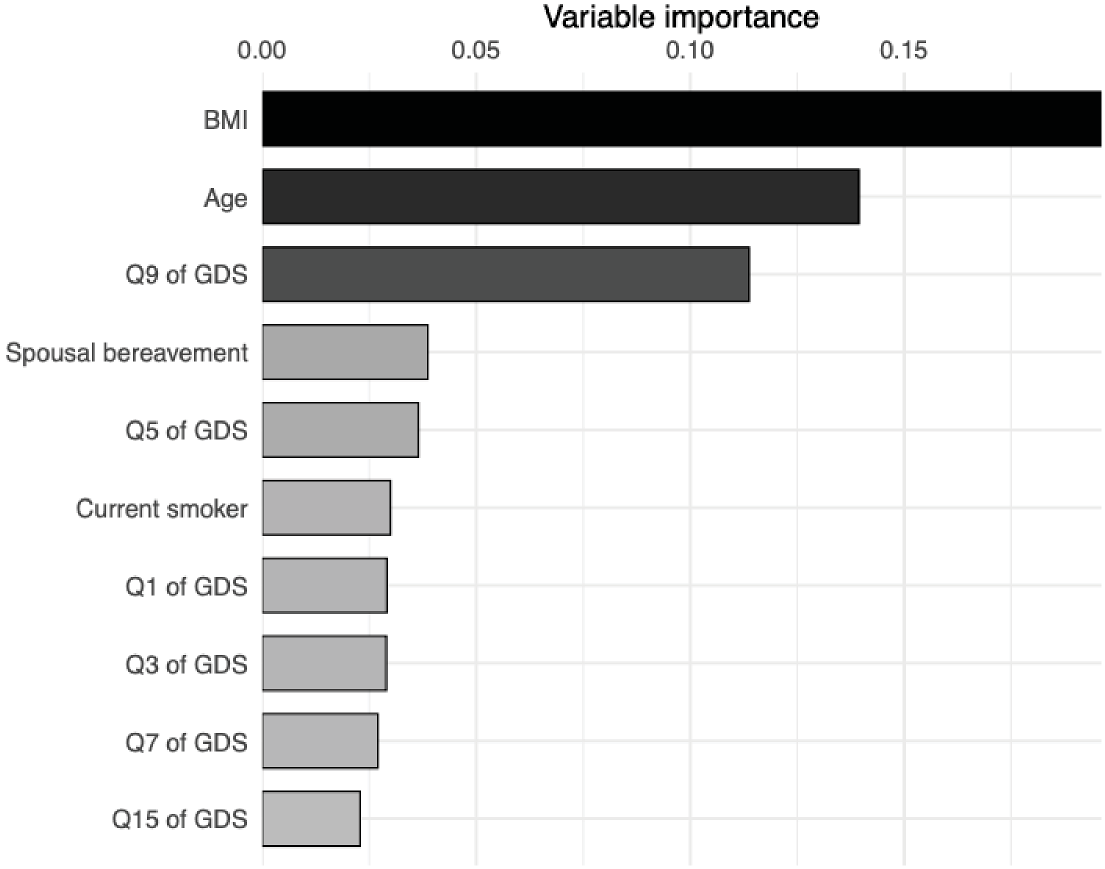

Abbreviations: BMI: body mass index; GDS: Geriatric Depression Scale

Variable importance was calculated as a weighted sum of the frequency with which each variable was selected for splitting at each depth in the causal forest. This variable importance plot highlights the top 10 variables.

"Q_X of GDS" denotes question number X of the Geriatric Depression Scale (GDS). For details on the GDS questions, please refer to eTable 1 (Geriatric Depression Scale-15).

© 2025 Takemura Y et al. *JAMA Network Open*

eTable 1. Geriatric depression scale 15 [3]

No.	Question	Answer
1.	Are you basically satisfied with your life?	Yes / No
2.	Do you feel pretty worthless the way you are now?	Yes / No
3.	Have you dropped many of your activities and interests?	Yes / No
4.	Do you feel that your life is empty?	Yes / No
5.	Do you often get bored?	Yes / No
6.	Are you in good spirits most of the time?	Yes / No
7.	Are you afraid that something bad is going to happen to you?	Yes / No
8.	Do you feel happy most of the time?	Yes / No
9.	Do you often feel helpless?	Yes / No
10.	Do you prefer to stay at home, rather than going out and doing new things?	Yes / No
11.	Do you feel you have more problems with memory than most people?	Yes / No
12.	Do you think it is wonderful to be alive?	Yes / No
13.	Do you feel full of energy?	Yes / No
14.	Do you feel that your situation is hopeless?	Yes / No
15.	Do you think that most people are better off than you are?	Yes / No

© 2025 Takemura Y et al. *JAMA Network Open*

eTable 2. Baseline characteristics of participants in both 2016 and 2019, and those followed from 2016, 2019, to 2022.

Variables [a]		Participants in 2016 to 2019 [b, c]			Participants in 2016, 2019 to 2022 [b, d]		
		No social participation in 2019 (N = 30,616)	Social participation in 2019 (N = 37,801)	SMD	No social participation in 2019 (N = 16,133)	Social participation in 2019 (N = 22,039)	SMD
Age (mean (SD)), years		72.90 (5.81)	73.10 (5.31)	0.035	72.11 (5.18)	72.60 (4.90)	0.097
Gender, N (%)	Men	16632 (54.3)	16186 (42.8)	0.232	8931 (55.4)	9544 (43.3)	0.243
	Women	13984 (45.7)	21615 (57.2)		7202 (44.6)	12495 (56.7)	
BMI (mean (SD))		22.99 (3.22)	22.82 (2.93)	0.054	23.09 (3.17)	22.84 (2.90)	0.08
Work.status, N (%)	Working	9308 (30.4)	8763 (23.2)	0.17	5310 (32.9)	5284 (24.0)	0.205
	Retired	15756 (51.5)	22123 (58.5)		8171 (50.6)	12994 (59.0)	
	Nonworking	1652 (5.4)	2175 (5.8)		784 (4.9)	1237 (5.6)	
	Missing	3900 (12.7)	4740 (12.5)		1868 (11.6)	2524 (11.5)	
Marital.status, N (%)	Married	23081 (75.4)	28651 (75.8)	0.15	12561 (77.9)	17108 (77.6)	0.155
	Widowed	4437 (14.5)	6680 (17.7)		2139 (13.3)	3685 (16.7)	
	Divorced	1530 (5.0)	1299 (3.4)		706 (4.4)	654 (3.0)	
	Unmarried	1065 (3.5)	776 (2.1)		504 (3.1)	411 (1.9)	
	Others	223 (0.7)	142 (0.4)		111 (0.7)	59 (0.3)	
	Missing	280 (0.9)	253 (0.7)		112 (0.7)	122 (0.6)	
Educational level, N (%)	<6 years	191 (0.6)	87 (0.2)	0.225	65 (0.4)	40 (0.2)	0.224
	6-9 years	9109 (29.8)	8000 (21.2)		4761 (29.5)	4608 (20.9)	

© 2025 Takemura Y et al. *JAMA Network Open*

	10-12 years	12915 (42.2)	16920 (44.8)		7012 (43.5)	10015 (45.4)	
	≥13 years	8041 (26.3)	12406 (32.8)		4141 (25.7)	7166 (32.5)	
	Others	135 (0.4)	206 (0.5)		59 (0.4)	122 (0.6)	
	Missing	225 (0.7)	182 (0.5)		95 (0.6)	88 (0.4)	
Annual household.income, US $, N (%)	<18 386	6673 (21.8)	6302 (16.7)	0.164	3188 (19.8)	3362 (15.3)	0.149
	18 386-36 771	11586 (37.8)	14966 (39.6)		6194 (38.4)	8789 (39.9)	
	>36 771	8642 (28.2)	12662 (33.5)		4876 (30.2)	7720 (35.0)	
	Missing	3715 (12.1)	3871 (10.2)		1875 (11.6)	2168 (9.8)	
Medical.history, N (%)	Hypertension	13447 (43.9)	15147 (40.1)	0.13	7118 (44.1)	8761 (39.8)	0.132
	Stroke	344 (1.1)	326 (0.9)		160 (1.0)	170 (0.8)	
	Heart diseases	1389 (4.5)	1440 (3.8)		670 (4.2)	801 (3.6)	
	Diabetes	1624 (5.3)	1774 (4.7)		860 (5.3)	1028 (4.7)	
	Hyperlipidemia	1384 (4.5)	2508 (6.6)		823 (5.1)	1600 (7.3)	
	Others[e]	11259 (36.8)	15100 (39.9)		5916 (36.7)	8831 (40.1)	
	Missing	1169 (3.8)	1506 (4.0)		586 (3.6)	848 (3.8)	
Smoking status, N (%)	Nonsmoker	16100 (52.6)	24192 (64.0)	0.253	8478 (52.6)	14109 (64.0)	0.251
	Past smoker	10046 (32.8)	10224 (27.0)		5420 (33.6)	6056 (27.5)	
	Current smoker	4005 (13.1)	2862 (7.6)		1995 (12.4)	1597 (7.2)	
	Missing	465 (1.5)	523 (1.4)		240 (1.5)	277 (1.3)	
Alcohol consumption, N (%)	Nondrinker	13991 (45.7)	18068 (47.8)	0.137	7279 (45.1)	10493 (47.6)	0.122
	Past drinker	3539 (11.6)	2863 (7.6)		1697 (10.5)	1570 (7.1)	
	Current drinker	12440 (40.6)	15960 (42.2)		6821 (42.3)	9472 (43.0)	
	Missing	646 (2.1)	910 (2.4)		336 (2.1)	504 (2.3)	

© 2025 Takemura Y et al. *JAMA Network Open*

IADL, N (%)	Independence	26915 (87.9)	35319 (93.4)	0.198	14442 (89.5)	20718 (94.0)	0.167
	Dependence	2982 (9.7)	1812 (4.8)		1316 (8.2)	958 (4.3)	
	Missing	719 (2.3)	670 (1.8)		375 (2.3)	363 (1.6)	
Unmarried or living alone, N (%)	No	22134 (72.3)	27729 (73.4)	0.034	12097 (75.0)	16600 (75.3)	0.019
	Yes	8202 (26.8)	9819 (26.0)		3924 (24.3)	5317 (24.1)	
	Missing	280 (0.9)	253 (0.7)		112 (0.7)	122 (0.6)	
Poor interaction with children, N (%)	No	21296 (69.6)	27364 (72.4)	0.062	11305 (70.1)	16066 (72.9)	0.063
	Yes	9001 (29.4)	10072 (26.6)		4678 (29.0)	5788 (26.3)	
	Missing	319 (1.0)	365 (1.0)		150 (0.9)	185 (0.8)	
Poor interaction with relatives, N (%)	No	14955 (48.8)	18881 (49.9)	0.025	8069 (50.0)	11213 (50.9)	0.018
	Yes	15160 (49.5)	18370 (48.6)		7849 (48.7)	10552 (47.9)	
	Missing	501 (1.6)	550 (1.5)		215 (1.3)	274 (1.2)	
Poor interaction with friends, N (%)	No	16162 (52.8)	30216 (79.9)	0.601	8788 (54.5)	17746 (80.5)	0.581
	Yes	13629 (44.5)	7022 (18.6)		6980 (43.3)	4000 (18.1)	
	Missing	825 (2.7)	563 (1.5)		365 (2.3)	293 (1.3)	
Social participation in 2016, N (%)	No	20341 (66.4)	4374 (11.6)	1.704	10795 (66.9)	2481 (11.3)	1.73
	Yes	5810 (19.0)	31587 (83.6)		3082 (19.1)	18574 (84.3)	
	Missing	4465 (14.6)	1840 (4.9)		2256 (14.0)	984 (4.5)	

© 2025 Takemura Y et al. *JAMA Network Open*

Abbreviations: SMD: standardized mean difference, SD: standard deviation, BMI: body mass index, IADL: instrumental activities of daily living

[a] Continuous variables are presented as means and standard deviation (SD). Categorical variables are presented as the number of individuals and the percentages (%).

[b] 45.1% of participants in the survey in both 2016 and 2019 did not participate the survey in 2022.

[c] Among the participants from 2016 to 2019, data on social participation in 2019 were missing for 8,816 individuals.

[d] Among the participants from 2016, 2019, and 2022, data on social participation in 2019 were missing for 4,255 individuals.

[e.] other medical history includes pulmonary diseases, gastrointestinal diseases, kidney diseases, musculoskeletal disorders, trauma (such as falls and fractures), cancer, hematologic and immunologic disorders, ophthalmologic conditions, and otologic conditions.

© 2025 Takemura Y et al. *JAMA Network Open*

eTable 3. Comparison of characteristics among five subgroups based on quintiles of the CATE distribution.

Variables		Q1 [a] N=1115	Q2 [a] N=1114	Q3 [a] N=1115	Q4 [a] N=1114	Q5 [a] N=1115
Age (mean (SD)), years		68.40 (3.04)	70.85 (3.95)	73.02 (4.77)	74.50 (4.92)	75.48 (5.59)
Gender, N (%)	Men	473 (42.4)	608 (54.6)	668 (59.9)	659 (59.2)	506 (45.4)
	Women	642 (57.6)	506 (45.4)	447 (40.1)	455 (40.8)	609 (54.6)
BMI (mean (SD), kg/m2		23.77 (3.04)	23.03 (3.00)	22.89 (3.07)	22.85 (2.86)	23.16 (3.08)
Work status, N (%)	Working	508 (45.6)	422 (37.9)	395 (35.4)	324 (29.1)	332 (29.8)
	Retired	549 (49.2)	645 (57.9)	665 (59.6)	724 (65.0)	666 (59.7)
	Nonworking	58 (5.2)	47 (4.2)	55 (4.9)	66 (5.9)	117 (10.5)
Marital status, N (%)	Married	1054 (94.5)	981 (88.1)	894 (80.2)	844 (75.8)	728 (65.3)
	Widowed	23 (2.1)	62 (5.6)	139 (12.5)	210 (18.9)	333 (29.9)
	Divorced	20 (1.8)	45 (4.0)	42 (3.8)	30 (2.7)	30 (2.7)
	Unmarried	16 (1.4)	24 (2.2)	37 (3.3)	26 (2.3)	18 (1.6)
	Others	2 (0.2)	2 (0.2)	3 (0.3)	4 (0.4)	6 (0.5)
Educational level, N (%)	<6 years	1 (0.1)	0 (0.0)	2 (0.2)	3 (0.3)	10 (0.9)
	6-9 years	209 (18.7)	280 (25.1)	310 (27.8)	360 (32.3)	386 (34.6)
	10-12 years	562 (50.4)	512 (46.0)	496 (44.5)	428 (38.4)	455 (40.8)
	≥13 years	335 (30.0)	316 (28.4)	300 (26.9)	321 (28.8)	255 (22.9)

© 2025 Takemura Y et al. *JAMA Network Open*

	Others	8 (0.7)	6 (0.5)	7 (0.6)	2 (0.2)	9 (0.8)
Annual household income, US $, N (%)	≤18,386	124 (11.1)	164 (14.7)	234 (21.0)	264 (23.7)	282 (25.3)
	18,386 – 36,771	467 (41.9)	511 (45.9)	511 (45.8)	493 (44.3)	437 (39.2)
	≥36,771	524 (47.0)	439 (39.4)	370 (33.2)	357 (32.0)	396 (35.5)
Medical history, N (%)	Hypertension	427 (38.3)	461 (41.4)	454 (40.7)	524 (47.0)	609 (54.6)
	Stroke	2 (0.2)	12 (1.1)	11 (1.0)	6 (0.5)	8 (0.7)
	Heart diseases	45 (4.0)	50 (4.5)	52 (4.7)	40 (3.6)	40 (3.6)
	Diabetes	66 (5.9)	57 (5.1)	67 (6.0)	57 (5.1)	45 (4.0)
	Hyperlipidemia	76 (6.8)	58 (5.2)	53 (4.8)	59 (5.3)	57 (5.1)
	Others[b]	499 (44.8)	476 (42.7)	478 (42.9)	428 (38.4)	356 (31.9)
Smoking status, N (%)	Nonsmoker	718 (64.4)	601 (53.9)	595 (53.4)	559 (50.2)	692 (62.1)
	Past smoker	229 (20.5)	345 (31.0)	403 (36.1)	487 (43.7)	365 (32.7)
	Current smoker	168 (15.1)	168 (15.1)	117 (10.5)	68 (6.1)	58 (5.2)
Alcohol consumption, N (%)	Nondrinker	595 (53.4)	503 (45.2)	449 (40.3)	461 (41.4)	574 (51.5)
	Past drinker	62 (5.6)	92 (8.3)	139 (12.5)	128 (11.5)	98 (8.8)
	Current drinker	458 (41.1)	519 (46.6)	527 (47.3)	525 (47.1)	443 (39.7)
IADL, N (%)	Independence	1091 (97.8)	1044 (93.7)	1025 (91.9)	1027 (92.2)	1016 (91.1)
	Dependence	24 (2.2)	70 (6.3)	90 (8.1)	87 (7.8)	99 (8.9)
Unmarried or living alone, N (%)	No	1041 (93.4)	961 (86.3)	858 (77.0)	803 (72.1)	695 (62.3)

© 2025 Takemura Y et al. *JAMA Network Open*

	Yes	74 (6.6)	153 (13.7)	257 (23.0)	311 (27.9)	420 (37.7)
Poor interaction with children, N (%)	No	873 (78.3)	811 (72.8)	769 (69.0)	755 (67.8)	874 (78.4)
	Yes	242 (21.7)	303 (27.2)	346 (31.0)	359 (32.2)	241 (21.6)
Poor interaction with relatives, N (%)	No	618 (55.4)	531 (47.7)	531 (47.6)	571 (51.3)	704 (63.1)
	Yes	497 (44.6)	583 (52.3)	584 (52.4)	543 (48.7)	411 (36.9)
Poor interaction with friends, N (%)	No	835 (74.9)	796 (71.5)	734 (65.8)	707 (63.5)	746 (66.9)
	Yes	280 (25.1)	318 (28.5)	381 (34.2)	407 (36.5)	369 (33.1)
Social participation in 2016	No	515 (46.2)	502 (45.1)	490 (43.9)	463 (41.6)	450 (40.4)
	Yes	600 (53.8)	612 (54.9)	625 (56.1)	651 (58.4)	665 (59.6)

Abbreviations: CATE: conditional average treatment effect; SD: standard deviation; BMI: body mass index; IADL: instrumental activities of daily living.

[a] Q1 is the lowest quintile and Q5 is the highest quintile of the estimated CATE. In other words, as the categories progress from Q1 to Q5, they indicate groups that derive greater mental health benefits from social participation.

[b.] other medical history includes pulmonary diseases, gastrointestinal diseases, kidney diseases, musculoskeletal disorders, trauma (such as falls and fractures), cancer, hematologic and immunologic disorders, ophthalmologic conditions, and otologic conditions.

© 2025 Takemura Y et al. *JAMA Network Open*

eReferences

1. Fujihara S, Tsuji T, Miyaguni Y, et al. Does Community-Level Social Capital Predict Decline in Instrumental Activities of Daily Living? A JAGES Prospective Cohort Study. *Int J Environ Res Public Health*. 2019;16(5):828. doi:10.3390/ijerph16050828

2. Saito M, Kondo K, Ojima T, Hirai H, JAGES group. [Criteria for social isolation based on associations with health indicators among older people. A 10-year follow-up of the Aichi Gerontological Evaluation Study]. *Nihon Koshu Eisei Zasshi*. 2015;62(3):95-105. doi:10.11236/jph.62.3_95

3. Burke WJ, Roccaforte WH, Wengel SP. The short form of the Geriatric Depression Scale: a comparison with the 30-item form. *J Geriatr Psychiatry Neurol*. 1991;4(3):173-178. doi:10.1177/089198879100400310
