# Supplementary material for: Social Participation and Depressive Symptoms Among Older Adults
Source: JAMA Netw Open. 2025 Sep 8;8(9):e2530523. doi: 10.1001/jamanetworkopen.2025.30523 (PMC12418130; doi:10.1001/jamanetworkopen.2025.30523)
Supplement: Supplement 2. — Data Sharing Statement [file jamanetwopen-e2530523-s002.pdf]

Data Sharing Statement

Takemura. Social Participation and Depressive Symptoms Among Older Adults. *JAMA Netw Open*. Published September 08, 2025. doi:10.1001/jamanetworkopen.2025.30523

Data
Data available: Yes
Data types: Deidentified participant data
How to access data: The data that support the findings of this study are available from the Japan Gerontological Evaluation Study (https://www.jages.net/About-Jages/); however, we used these under license for the current study, and so the data are not publicly available.
When available: With publication

Supporting Documents
Document types: None

Additional Information
Who can access the data: researchers whose proposed use of the data has been approved
Types of analyses: for a specified purpose
Mechanisms of data availability: with a signed data access agreement
